# An Adaptive Vehicle Detection Model for Traffic Surveillance of Highway Tunnels Considering Luminance Intensity

**DOI:** 10.3390/s24185912

**Published:** 2024-09-12

**Authors:** Yongke Wei, Zimu Zeng, Tingquan He, Shanchuan Yu, Yuchuan Du, Cong Zhao

**Affiliations:** 1 Department of Geotechnical Engineering, College of Civil Engineering, Tongji University, Shanghai 200092, China; 2280096@tongji.edu.cn; 2 State Key Laboratory for Disaster Reduction in Civil Engineering, Tongji University, Shanghai 200092, China; 3 Guangxi New Development Transportation Group Co., Ltd., Nanning 530029, China; hetq@bgigc.com; 4 Key Laboratory of Road and Traffic Engineering of the Ministry of Education, Tongji University, Shanghai 201804, China; 2331673@tongji.edu.cn (Z.Z.); ycdu@tongji.edu.cn (Y.D.); 5 School of Computing and Artificial Intelligence, Southwest Jiaotong University, Chengdu 611756, China; 6 Engineering Research Center of Sustainable Urban Intelligent Transportation, Ministry of Education, Chengdu 611756, China; 7 National Engineering and Research Center for Mountainous Highways, China Merchants Chongqing Communications Research Design Institute Co., Ltd., Chongqing 400067, China; yushanchuan@cmhk.com

**Keywords:** vehicle detection, traffic surveillance, deep learning, abnormal luminance, highway tunnels

## Abstract

Vehicle detection is essential for road traffic surveillance and active safety management. Deep learning methods have recently shown robust feature extraction capabilities and achieved improved detection results. However, vehicle detection models often perform poorly under abnormal lighting conditions, especially in highway tunnels. We proposed an adaptive vehicle detection model that accounts for varying luminance intensities to address this issue. The model categorizes the image data into abnormal and normal luminance scenarios. We employ an improved CycleGAN with edge loss as the adaptive luminance adjustment module for abnormal luminance scenarios. This module adjusts the brightness of the images to a normal level through a generative network. Finally, YOLOv7 is utilized for vehicle detection. The experimental results demonstrate that our adaptive vehicle detection model effectively detects vehicles under abnormal luminance scenarios in highway tunnels. The improved CycleGAN can effectively mitigate edge generation distortion. Under abnormal luminance scenarios, our model achieved a 16.3% improvement in precision, a 1.7% improvement in recall, and a 9.8% improvement in mAP_0.5 compared to the original YOLOv7. Additionally, our adaptive luminance adjustment module is transferable and can enhance the detection accuracy of other vehicle detection models.

## 1. Introduction

In today’s traffic systems, numerous modules rely on vehicle detection [1,2,3]. For example, applications such as traffic surveillance [4,5] and active safety management require specific vehicle detection information [6,7]. However, vehicle detection research still encounters practical challenges, such as limited feature extraction capabilities and abnormal luminance scenarios.

Consequently, vehicle detection using deep learning has become a research hot spot [8,9]. Vehicle detection algorithms based on deep learning can be broadly classified into three categories: traditional feature-based approaches, sliding window-based detection, and deep learning-based methods. Traditional feature-based approaches [10,11,12] rely on handcrafted features and rule-based systems to detect vehicles. These methods often struggle with occlusion and complex backgrounds, leading to lower accuracy and robustness in real-world scenarios. Despite their simplicity, their sensitivity to environmental factors limits their applicability in dynamic traffic conditions. Sliding window-based detection methods [13,14] scan images with a fixed-size window to detect objects. While this approach can be more effective than traditional methods, it still suffers from high computational costs and inefficiencies. The methods need to process multiple windows across different scales and positions, resulting in slow detection speeds, making them unsuitable for real-time applications. In contrast, deep learning-based object detection algorithms have demonstrated superior performance in vehicle detection tasks [15,16,17]. These methods leverage the power of convolutional neural networks (CNNs) to automatically learn and extract features from data, significantly improving detection accuracy and robustness. Deep learning-based methods can be divided into two main categories: two-stage and single-stage approaches. Two-stage methods, such as R-CNN [18], first generate candidate regions and then classify them. However, the main drawback of these methods is their computational redundancy, resulting in slow testing speeds that impede real-time updates. To address the limitations of these two-stage methods, single-stage approaches like YOLO (You Only Look Once) [19] have been developed. These end-to-end detection models directly predict bounding boxes and classes from the entire image in a single pass, which significantly reduces computational complexity and improves detection speed.

While current vehicle detection algorithms have achieved impressive performance on many public datasets, they still struggle with varying luminance conditions in real-world scenarios [20]. Particularly in highway tunnels, the cameras are frequently affected by the headlights of passing vehicles [21]. This results in varying luminance intensities in consecutive image frames, which could cause missed detections [22,23]. Several studies have proposed illumination-adaptive detection models that adjust the overall image luminance by extracting and normalizing luminance values [24,25,26]. However, these approaches assume a uniform change in luminance across the entire image, which is rarely the case in real-world environments. When there are sudden luminance changes, the brightness of objects and their surroundings does not change uniformly. To address this issue, we should consider the heterogeneous nature of luminance changes across different regions of an image.

Research has demonstrated that generative models for style transfer can effectively modify different elements within an image [27,28]. Building on this foundation, we propose an adaptive vehicle detection model for traffic surveillance of highway tunnels considering luminance intensity. Our model incorporates an advanced CycleGAN-based adaptive luminance adjustment module. This module processes the images before they are fed into the vehicle detection module. It fine-tunes the luminance intensity of various parts of the image. This step could effectively minimize the impact of luminance differences on vehicle detection accuracy. By aligning the luminance distribution more closely with normal luminance scenarios, our methods significantly enhance the accuracy and robustness of vehicle detection.

We highlight the notable innovations of our model:

(1) Adaptive Vehicle Detection Model: We propose an adaptive vehicle detection model that specifically considers the luminance intensity inside highway tunnels. This approach achieves high-accuracy vehicle detection in tunnel environments, effectively addressing the challenges posed by abnormal luminance scenarios.

(2) Refined CycleGAN-Based Adaptive Luminance Adjustment Module: We introduce an advanced luminance adjustment module based on CycleGAN [28], which incorporates an edge loss mechanism. The edge loss mechanism ensures that the boundaries of vehicles are accurately maintained during the transformation process, resulting in improved detection performance and visual fidelity of the adjusted images.

(3) Extensibility and Integration: We demonstrate the extensibility of the proposed adaptive luminance adjustment module. This module can be seamlessly integrated into various vehicle detection models, enhancing their performance under different luminance intensities.

## 2. Literature Review

### 2.1. Traditional Vehicle Detection Algorithms

Traditional vehicle detection algorithms primarily consist of feature-based methods [29] and sliding window-based methods [13]. In feature-based vehicle detection, widely used feature extraction techniques include Haar features, wavelet transforms, histogram of oriented gradients (HOG) [30,31], and scale-invariant feature transform (SIFT) [32]. These feature extraction methods form the foundation of vehicle detection by capturing the shape, edges, and texture information of vehicles. For instance, HOG captures the gradient orientation of the image, which is particularly effective in detecting objects with defined edges and textures like vehicles. SIFT, on the other hand, provides robust feature descriptors that are invariant to scale and rotation, enhancing the detection capability under varying conditions.

Sliding window-based vehicle detection is another significant traditional method [14]. This approach detects potential vehicles by progressively scanning the image with windows of different sizes. The sliding window technique often integrates feature extraction with classifiers. A notable example is the detection framework that combines HOG features with a support vector machine (SVM) [33]. This combination leverages the robust feature extraction capability of HOG and the classification power of SVM to identify vehicles accurately. However, while sliding window-based methods improve detection accuracy, they require extensive processing for multiple window scans and feature evaluations, which makes them more computationally intensive compared to feature-based methods.

Although traditional vehicle detection algorithms have been gradually outperformed by deep learning methods, some of the traditional methods still hold value in specific scenarios. The traditional feature-based methods have lower computational requirements for feature extraction compared to deep learning algorithms, making them more advantageous in scenarios with limited computational resources.

### 2.2. Deep Learning-Based Vehicle Detection Algorithms

Deep learning-based object detection algorithms have made significant progress in vehicle detection in recent years. These algorithms are mainly divided into two-stage and single-stage methods.

Representative two-stage algorithms include the region-based convolutional neural network (R-CNN) series, such as R-CNN [18], Fast R-CNN [34], Faster R-CNN [35], and Mask R-CNN [36]. These algorithms first generate region proposals. They then perform precise object classification and bounding box regression on each proposal. The R-CNN series methods, using selective searches to generate region proposals, achieve high detection accuracy but have relatively low computational efficiency. Fast R-CNN integrates feature extraction and classification into a single network, improving detection speed. Faster R-CNN introduces the region proposal network (RPN), significantly enhancing detection efficiency. Mask R-CNN adds instance segmentation capabilities to Faster R-CNN, enabling pixel-level object segmentation in addition to object detection.

Single-stage methods mainly include the YOLO series [19] and the single-shot multiBox detector (SSD) [37]. The YOLO series treats object detection as a regression problem. It predicts bounding boxes and classes directly through a single neural network, resulting in high detection speeds [38]. YOLO achieved a good balance between speed and accuracy. YOLOv2 [39] and YOLOv3 [40] improved detection performance by introducing multi-scale prediction and deeper network structures. SSD uses multiple feature layers for multi-scale detection. This approach maintains high detection speeds while improving accuracy for small objects [41]. With the development of deep learning, various emerging deep learning methods can provide valuable references for vehicle detection research [42,43].

Despite the increasing accuracy of these methods in vehicle detection tasks, single-vehicle detection models struggle with varying luminance conditions. For example, in tunnel scenarios, cameras are easily affected by the headlights of passing vehicles. This results in varying luminance intensities in consecutive image frames, which can lead to missed detections [44].

### 2.3. Generative Adversarial Networks (GANs)

Generative adversarial networks (GANs) [45] have become a significant research direction in the field of generative models. GANs consist of a generator and a discriminator that compete against each other through adversarial training to generate realistic data. The generator aims to produce realistic samples from random noise, while the discriminator’s goal is to distinguish between real and generated samples. This adversarial process improves the quality of generated samples over time.

GANs have wide-ranging applications, including image generation, image inpainting, image super-resolution, style transfer, text generation, and video generation [46,47,48]. In the field of image generation, GANs can produce high-quality, realistic images, making significant contributions to unsupervised and semi-supervised learning. Deep convolutional GAN (DCGAN) [49] introduced CNNs, enhancing the quality and stability of generated images and broadening the application of GANs in image generation. StyleGAN [47] introduced style mixing and progressive growing mechanisms, further improving image generation quality, especially in realistic face generation. CycleGAN [28] excels in style transfer, achieving unsupervised image-to-image translation.

Currently, numerous studies have combined generative models with object detection. These studies aim to enhance detection performance through day-to-night transformations. Lin et al. [50] proposed AugGAN, a GAN-based data augmenter. AugGAN converts nighttime road environments to daytime ones, thereby improving vehicle detection accuracy at night. Liu et al. [51] introduced a high-precision vehicle detection algorithm. This algorithm uses a refined GAN to enhance vehicle features in nighttime images. Zhou et al. [52] proposed a new all-day vehicle detection framework. This framework incorporates the illumination-adaptive GAN (IA-GAN). The IA-GAN uses adjustable luminance vectors as input. It converts labeled daytime images into multiple nighttime images with varying illumination.

Although the generative models in these studies have improved detection accuracy, these models can be unstable. The generated results may deviate from real traffic conditions. Therefore, for vehicle detection tasks in traffic scenes, it is necessary to introduce more constraints into the models to enhance the realism of the generated images.

## 3. Methodology

**Figure 1:**
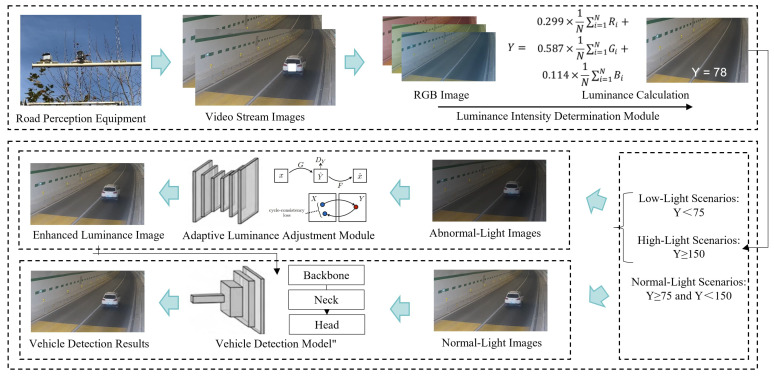
This is the framework for the adaptive vehicle detection model that considers tunnel luminance intensity. First, the luminance intensity determination module evaluates the luminance intensity of the images. If the luminance is either too dim or too bright, the images are input into the adaptive luminance adjustment module for luminance correction. After adjustment, the images are input into the vehicle detection model based on YOLOv7, which outputs the vehicle detection information.

This section introduces our adaptive vehicle detection model for traffic surveillance of highway tunnels considering luminance intensity. The framework of our model is illustrated in Figure 1. The model begins by acquiring video stream data from roadside facility detection systems. Once the video data are obtained, the luminance intensity determination module evaluates the luminance intensity of the captured frame images. It determines the processing path for the images based on their luminance levels. If the luminance is deemed normal, the images proceed directly to the subsequent vehicle detection module. If the luminance is either too dim or too bright, the images are input into the adaptive luminance adjustment module for correction. The adaptive luminance adjustment module employs an improved CycleGAN to generate images with normalized luminance intensity. Additionally, the adaptive luminance adjustment module also considers edge loss in the images. This preserves more of the original image’s object information, ensuring critical features are retained in the generated images. Finally, the processed images are input into the vehicle detection module based on YOLOv7. This module analyzes the images and outputs precise vehicle detection location information.

### 3.1. Luminance Intensity Determination Module

The color space *Y*$C_{r}$$C_{b}$ is commonly used by European TV systems. This module processes RGB data from video stream images and applies *Y*$C_{r}$$C_{b}$ color space theory to separate the image into luminance (*Y*) and chrominance ($C_{r}$$C_{b}$) components. In this color space, the *Y* component does not carry any color information, which made this color space more suitable for the luminance adjustment for our detection task. The luminance (*Y*) is calculated using the *Y*$C_{r}$$C_{b}$ color space formula, and the parameters in the formula are referenced from the research [53]:

(1) $$Y=\begin{array}{c} 0.299\times \frac{1}{N}\sum_{i=1}^{N}R_{i}+\\ 0.587\times \frac{1}{N}\sum_{i=1}^{N}G_{i}+\\ 0.114\times \frac{1}{N}\sum_{i=1}^{N}B_{i} \end{array}$$

where $R_{i}$, $G_{i}$, $B_{i}$ are the red, green, and blue values of the *i*-th pixel in the image, and *N* denotes the total number of pixels.

After calculating luminance (*Y*), the video stream images are categorized based on preset thresholds: abnormal luminance images and normal luminance images. The preset thresholds of luminance can be determined based on the specific vehicle detection task or selected according to the luminance distribution of the dataset in the actual scenario, using the 95th percentile and the 5th percentile as the upper and lower bounds for normal luminance, respectively. Then, the normal luminance images proceed directly to the vehicle detection module. However, abnormal luminance images undergo processing through the adaptive luminance adjustment module to normalize their luminance intensity. Subsequently, these adjusted images are input into the vehicle detection module for precise vehicle detection and localization.

### 3.2. Adaptive Luminance Adjustment Module Based on Improved CycleGAN

CycleGAN is widely adopted for image generation due to its effectiveness in style transformation, particularly in scenarios where paired training data are unavailable [28]. CycleGAN leverages two key concepts: adversarial learning and cycle consistency, enabling it to perform unpaired image-to-image translation effectively. We leverage this model as the foundation for adaptive luminance adjustment to address the challenges posed by varying lighting conditions in vehicle detection tasks.

**Figure 2:**
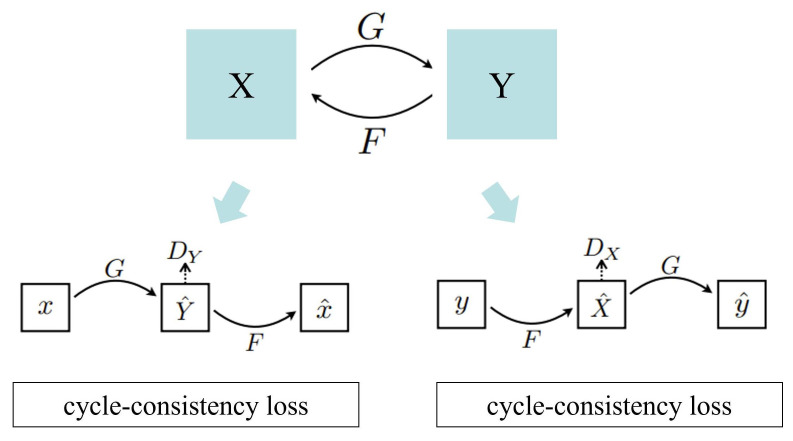
This is the model framework diagram of CycleGAN. The model consists of two generators (*G* and *F*) and two discriminators ($D_{X}$ and $D_{Y}$). Generator *G* maps the input image from one domain to the target domain, while Generator *F* converts it back to the original domain.

Figure 2 illustrates the principle of CycleGAN, which includes two generators (*G* and *F*) and two discriminators ($D_{X}$ and $D_{Y}$). The role of the generators is to learn the mapping between two different domains, such as images with abnormal luminance and those with normal luminance. Specifically, Generator *G* maps input images from domain *X* (e.g., images with abnormal luminance) to domain *Y* (e.g., images with normal luminance), while Generator *F* performs reverse mapping from domain *Y* back to domain *X*. The discriminators, $D_{X}$ and $D_{Y}$, are used to distinguish between real images from their respective domains and the generated images produced by the corresponding generators. The adversarial loss ensures that the generated images are indistinguishable from the real images in the target domain, promoting the realism of the generated images.

In addition to the adversarial loss, the cycle consistency loss plays a crucial role in the CycleGAN framework. The cycle consistency loss requires that an image from one domain, when translated to the other domain and then back again, should return to its original form. This loss is formulated as ||F(G(X))−X|| and ||G(F(Y))−Y||, ensuring that the mapping learned by the generators is robust and that the essential content and structure of the images are preserved during translation. By enforcing cycle consistency, the model mitigates the risk of arbitrary transformations that could lead to a loss of important image features. This dual-objective framework allows CycleGAN to perform high-quality image style transfer and luminance adjustment without the need for paired training data, which is particularly advantageous in real-world applications where obtaining such data can be challenging.

**Figure 3:**
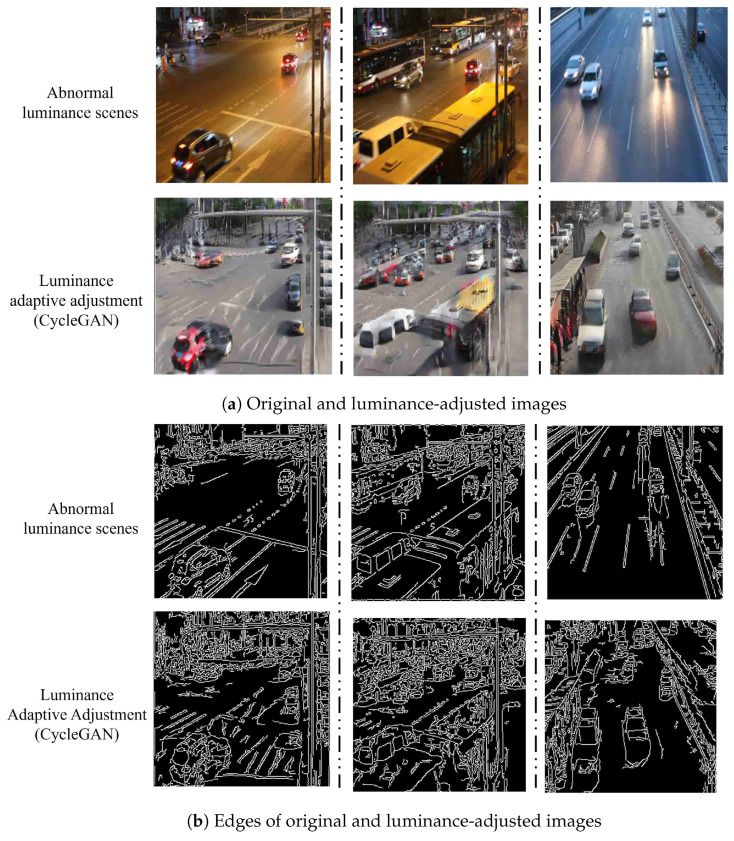
The example of luminance adjustment using the original CycleGAN. (**a**) shows the images of abnormal luminance scenes and their counterparts after luminance adjustment with the original CycleGAN. (**b**) shows the edges of the images of abnormal luminance scenes and their counterparts after luminance adjustment with the original CycleGAN.

However, experiments have revealed that CycleGAN may introduce blurred details and possibly create spurious edges when adapting luminance in tunnel images. Such artifacts can adversely impact the accuracy of vehicle detection models, as depicted in Figure 3.

Figure 3a shows images of abnormal luminance scenes and their corresponding versions after luminance adjustment using the original CycleGAN. While the images generated by the original CycleGAN demonstrate an improvement in luminance, they also contain some redundant vehicles. As seen in the edge results in Figure 3b, there are noticeable differences in the object edges in the images produced by the original CycleGAN. This discrepancy may be due to the lack of constraints on the object edges in the model. Therefore, we need to make adjustments to the model to ensure its effectiveness. The model should not only normalize the luminance of the images but also generate images where the object edges closely align with those in the original images.

Therefore, we modified the loss function by introducing an edge loss term to quantify the difference in edge features between the generated and original images. Specifically, edge images were generated for both the original and generated images using the Canny edge extraction algorithm [54,55]. The Canny edge detection algorithm is a multi-stage process used to identify the edges in an image, which are significant changes in intensity. It begins by applying a Gaussian filter to smooth the image and reduce noise. Then, it computes the gradient magnitude and direction using edge detection operators, such as the Sobel operator. Non-maximum suppression is then performed to thin out the edges, retaining only local maxima. Finally, double thresholding is applied to distinguish between strong, weak, and non-relevant edges, followed by edge tracking through hysteresis to connect weak edges that are connected to strong ones, ensuring a clean and continuous edge representation in the final output.

Then, the edge similarity between images is measured using the L2 loss (mean squared error, MSE) during the loss calculation process. The loss function is as follows:

(2) $$L_{\mathrm{edge}\;}=\sum {∥\mathrm{Edge}\left(I_{\mathrm{original}\;}\right)-\mathrm{Edge}\left(I_{\mathrm{generated}\;}\right)∥}^{2}$$

(3) $$\mathrm{Loss}=Loss_{\mathrm{gan}}+Loss_{\mathrm{cycle}}+Loss_{\mathrm{identity}}+Loss_{\mathrm{edge}}$$

In the formula, $I_{\mathrm{original}}$ and $I_{\mathrm{generated}}$ represent the original image and the generated image, respectively. Edge is the edge detection function, which uses the Canny edge extraction algorithm. $L_{\mathrm{gan}}$ is the GAN loss, which measures the difference between the generated images and the real images. $L_{\mathrm{cycle}}$ is the cycle consistency loss, which ensures that the generated images can be transformed back to the original images by the generator’s inverse transformation. $L_{\mathrm{identity}}$ is the identity loss, which constrains the generator to produce identical images when processing images already in the target domain. $L_{\mathrm{edge}}$ represents the edge loss between the original and generated images.

In the framework of our adaptive vehicle detection model, the adaptive luminance adjustment module accepts images with abnormal luminance intensity as the input. It generates corresponding images with normalized luminance intensity, effectively preserving the original image’s edge information. These processed images are subsequently utilized by the vehicle detection modules.

### 3.3. Vehicle Detection Module Based on YOLOv7

**Figure 4:**
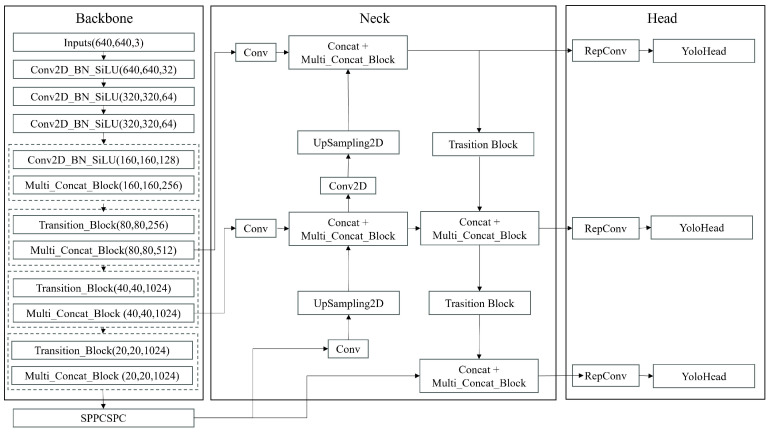
This is the model architecture of YOLOv7.

The YOLO series models are highly regarded in the field of object detection and are widely adopted by researchers due to their exceptional performance across diverse domains [19]. This study selected the YOLOv7 model from the YOLO series. YOLOv7 [56] comprises three main modules: Backbone, Neck, and Head. The Backbone module of the YOLOv7 network is primarily constructed using convolutional layers, E-ELAN modules, MPConv modules, and an SPPCSPC module. The SPPCSPC module integrates multiple parallel MaxPool operations within the convolution process to mitigate image distortion and other issues caused by image processing operations. In the Neck module, YOLOv7 utilizes a PAFPN (Path Aggregation Feature Pyramid Network) structure. The multiple effective feature layers obtained in the Backbone module are fused in this section. The purpose of this feature fusion is to integrate information from different scales. The Head module makes the final detection outputs for the network and YOLOv7 adds auxiliary heads to the network to obtain better results. The detection outputs of YOLOv7 include object coordinates, category probabilities, and detection confidence scores. The model architecture is depicted in Figure 4.

The training process of YOLOv7 adopts an iterative approach. In each training batch, the model predicts bounding boxes for objects within images, calculates the loss by comparing these predictions to the ground truth bounding boxes, and updates the model weights using backpropagation and optimization algorithms.

The loss function of the YOLOv7 model consists of three main components: localization loss $L_{\mathrm{Localization}}$, confidence loss $L_{\mathrm{Confidence}}$, and classification loss $L_{\mathrm{Classification}}$. These loss functions are designed to optimize the positional accuracy, confidence level, and category classification accuracy simultaneously in object detection tasks.

(4) $$L_{\mathrm{YOLO}}=L_{\mathrm{Localization}}+L_{\mathrm{Confidence}}+L_{\mathrm{Classification}}$$

Localization loss employs the Complete Intersection over Union (CIoU) loss function to quantify the disparity between the model’s predicted bounding boxes and the ground truth positions. Confidence loss assesses the model’s certainty in predicting object presence within a bounding box, often formulated through cross-entropy loss. Classification loss ensures the precise classification of detected objects into their respective categories, evaluated by cross-entropy loss comparing predicted and ground truth categories. The computation of these loss functions follows these formulas:

(5) $$L_{\mathrm{Localization}}=1-IoU+\frac{\rho^{2}\left(b,b_{\mathrm{gt}}\right)}{c^{2}}+\beta v$$

(6) $$\beta =\frac{v}{1-\mathrm{IoU}+v}$$

(7) $$v=\frac{4}{\pi^{2}}\cdot {\left(\arctan\;\frac{w_{\mathrm{gt}}}{\sqrt{h_{\mathrm{gt}}}}-\arctan\;\frac{w}{h}\right)}^{2}$$

(8) $$\begin{array}{cc} \; & L_{\mathrm{Confidence}}=-\sum_{i=0}^{S^{2}}\sum_{j=0}^{B}I_{i,j}^{\mathrm{obj}\;}\left[C_{i}^{j}\log\left({\hat{C}}_{i}^{j}\right)+\left(1-C_{i}^{j}\right)\cdot \log\left(1-{\hat{C}}_{i}^{j}\right)]-\right.\\ \; & \sum_{i=0}^{s^{2}}\sum_{j=0}^{B}I_{i,j}^{\mathrm{noobj}}\left[C_{i}^{j}\log\left({\hat{C}}_{i}^{j}\right)+\left(1-C_{i}^{j}\right)\log\left(1-{\hat{C}}_{i}^{j}\right)\right] \end{array}$$

(9) $$\begin{array}{c} L_{\mathrm{Classification}\;}=-\sum_{i=0}^{s^{2}}I_{i,j}^{\mathrm{obj}\;}\sum_{c=1}^{c}\left[p_{i}^{j}\log\left({\hat{p}}_{i}^{j}\right)+\left(1-p_{i}^{j}\right)\log\left(1-{\hat{p}}_{i}^{j}\right)\right] \end{array}$$

In the formula, w,h are the width and height of the predicted box. $w_{\mathrm{gt}},h_{\mathrm{gt}}$ are the width and height of the ground truth box. ρ,β,v are parameters used to adjust the localization loss. $\rho^{2}\left(b,b_{\mathrm{gt}}\right)$ is the Euclidean distance between the center points of the predicted box and the ground truth box. *c* is the diagonal distance of the minimum closed region containing the predicted and ground truth boxes. IoU is the Intersection over the Union between the predicted box and the ground truth box. $S^{2}$ and *B* are the scale of the feature map and the number of anchor boxes, respectively. $I_{i,j}^{\mathrm{obj}\;},I_{i,j}^{\mathrm{noobj}\;}$ indicate whether the *j*-th bounding box in cell *i* is responsible for predicting an object. *C* is the number of categories. These formulas encapsulate the mathematical framework used to optimize the YOLOv7 model during training, ensuring robust performance in object detection tasks.

In our vehicle detection model framework, this module takes images as input and outputs the positions of vehicles detected within those images.

## 4. Experiments

To validate the effectiveness and accuracy of the proposed adaptive vehicle detection model incorporating luminance adaptation, multiple sets of experiments were conducted. Firstly, efficacy validation experiments were performed to demonstrate that our model enhances detection accuracy in abnormal lighting conditions compared to traditional vehicle detection models. Secondly, the model was integrated into other vehicle detection frameworks to assess its scalability.

The experiments were conducted on an Ubuntu 20.04 operating system, utilizing a single RTX 3080 GPU and an Intel(R) Core(TM) i9-10850K CPU. The PyTorch 2.0.1 framework (developed by Facebook, Menlo Park, CA, USA) was employed, leveraging CUDA 11.7 and cuDNN 8500 (sourced from NVIDIA, Santa Clara, CA, USA) for accelerated model training. In this study, the YOLOv7 model and the luminance adaptation module were trained with 200 and 100 epochs, respectively, using the Adam optimizer and cosine annealing learning rate decay. The batch size of YOLOv7 was 16, and the batch size of the luminance adaptation module was 4.

### 4.1. Dataset

**Figure 5:**
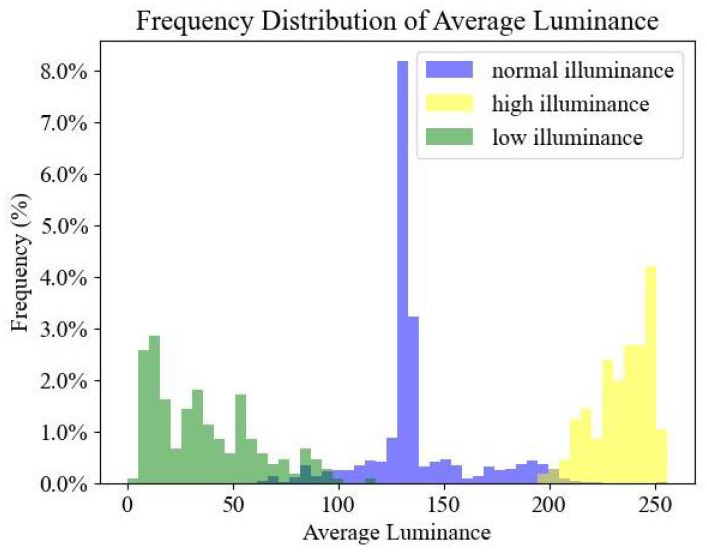
The luminance intensity distribution of the dataset.

For the training and validation of the adaptive luminance adjustment module, we compiled a diverse dataset consisting of images from both tunnel and natural environments. Images were captured inside tunnels using cameras, covering a range of conditions, including nighttime and daytime scenes, as well as some instances of overexposure. Additionally, we randomly sampled images with varying luminance levels from the LOw-Light dataset (LOL) [57]. This combination of tunnel-captured and LOL-sourced images created a comprehensive dataset for our study. The datasets include scenarios of high-luminance scenarios (1150 images), low-luminance scenarios (1150 images), and normal-luminance scenarios (2300 images). The dataset was split into an 8:2 ratio for training and testing. The luminance intensity distribution of the dataset is shown in Figure 5.

For the vehicle detection task, video frame images from various luminance scenarios inside highway tunnels were collected, annotated with vehicle positions, and augmented using methods such as rotation and offset. The augmented dataset consisted of 2600 pairs of data, including 800 pairs under high-luminance scenarios, 800 pairs under low-luminance scenarios, and 1000 pairs under normal-luminance scenarios. The dataset was split into an 8:2 ratio for training and testing.

### 4.2. Evaluation Metrics

For the adaptive luminance adjustment module, this study utilized the Fréchet inception distance (FID) and Kernel inception distance (KID) metrics to evaluate the distributional differences between generated and real images.

- FID(Frechet inception distance): Measures feature space similarity between real and generated images using the Frechet distance between multivariate Gaussian distributions:
  (10) $$\mathrm{FID}=∥\mu_{r}-\mu_{g}{∥}_{2}^{2}+\mathrm{Tr}\left(\Sigma_{r}+\Sigma_{g}-2{\left(\Sigma_{r}\Sigma_{g}\right)}^{1/2}\right)$$
  where $\mu_{r},\Sigma_{r}$ are the mean vector and covariance matrix of features from real images, and $\mu_{g},\Sigma_{g}$ are those from generated images.

For the vehicle detection task, this study employed metrics such as Accuracy, F1 Score, Precision, and Recall to evaluate the performance of the model.

- Precision: The ratio of true positive results to the sum of true positive and false positive results, indicating the accuracy of positive predictions.
- Recall: The ratio of true positive results to the sum of true positive and false negative results, measuring the ability to correctly identify positive instances.
- mAP: The metric used in object detection to evaluate a model’s accuracy. It calculates the mean of the Average Precision (AP) scores across all classes, with AP measuring the precision–recall curve for each class. A higher mAP value signifies better performance in both detecting and localizing objects. Specifically, mAP_0.5 refers to the mAP calculated with an Intersection over Union (IoU) threshold of 0.5.

### 4.3. Comparison Model

For the comparison experiments, we chose some common and widely used models. These models have different structures and are all widely used in image generation and vehicle detection. They are briefly described as follows:

- CycleGAN: This model enables unpaired image-to-image translation by employing cycle-consistency loss, ensuring that the generated images retain the key features and structures of the original images. It has revolutionized tasks such as style transfer, object transfiguration, and photo enhancement without the need for paired datasets.
- YOLOv7: This model improves upon its predecessors by incorporating advanced techniques such as CSPDarknet as a backbone and spatial pyramid pooling. These enhancements allow YOLOv7 to achieve high detection speeds and accuracy.
- Faster R-CNN: This model utilizes a region proposal network (RPN) to generate candidate object proposals, which are then refined using a convolutional neural network (CNN).
- SSD: This model eliminates the need for a separate region proposal stage by directly predicting object categories and bounding box offsets from feature maps at multiple scales.

### 4.4. Result Analysis

#### 4.4.1. Validation of the Modification of Adaptive Luminance Adjustment Module

**Figure 6:**
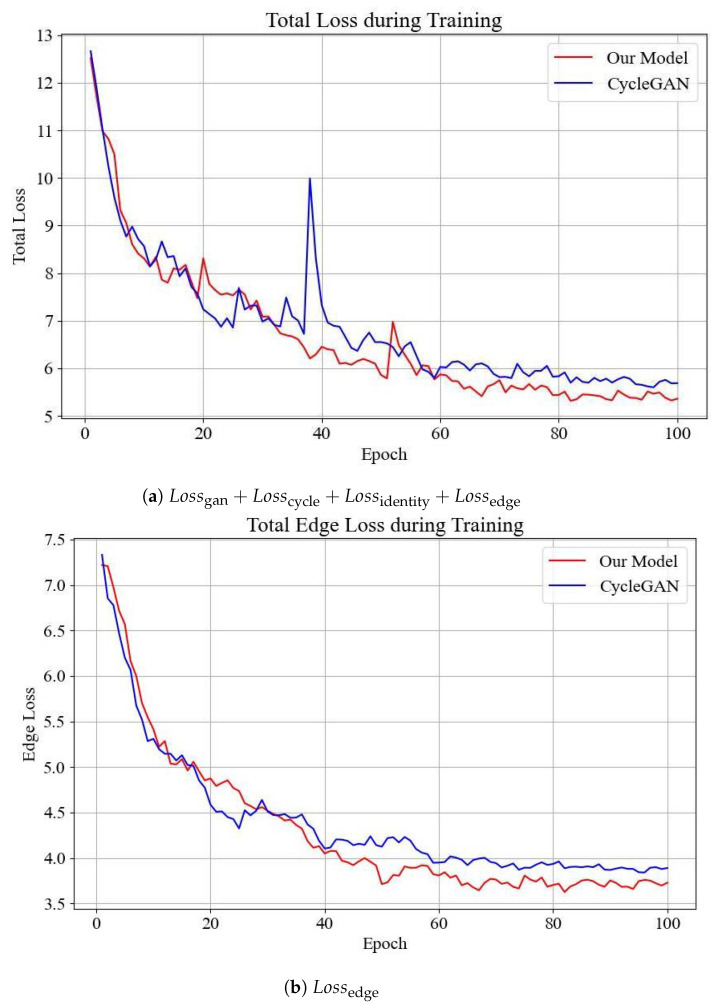
The loss during the model training. (**a**) $Loss_{\mathrm{gan}}+Loss_{\mathrm{cycle}}+Loss_{\mathrm{identity}}+Loss_{\mathrm{edge}}$ during the model training. (**b**) $Loss_{\mathrm{edge}}$ during the model training.

To quantitatively assess the impact of edge loss on luminance adjustment within the adaptive luminance adjustment module, we trained both our model and the original CycleGAN on the dataset. Figure 6a presents the evolution of the total loss for the original CycleGAN and our model during training. The results reveal that incorporating edge loss enables our model to achieve a lower overall loss compared to the original CycleGAN after training. Additionally, Figure 6b shows a consistent decrease in edge loss during training, which eventually stabilizes. This finding indicates that the inclusion of edge loss not only maintains the generation quality in terms of style and pixel accuracy but also significantly improves the preservation of edge information in our model.

We employed the FID metric to evaluate the model’s performance before and after integrating edge loss. The experimental results, presented in Table 1, demonstrate that the inclusion of edge loss significantly enhances image generation quality.

**Figure 7:**
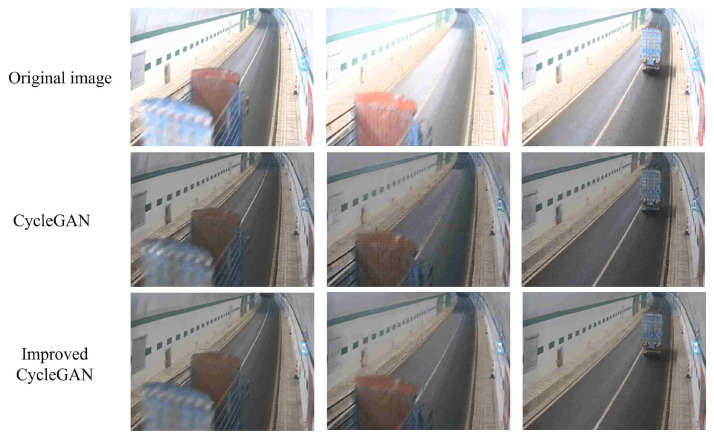
These are the visualization results of the generated images in the experiment.

Specifically, for the task of adjusting luminance from high intensity to normal intensity, the FID score decreased by 7.268%. Similarly, for the task of adjusting luminance from low intensity to normal intensity, the FID score decreased by 3.540%. These reductions in FID scores indicate that the integration of edge loss in the improved CycleGAN effectively improves the realism and quality of the generated images. The representative visual results of these experiments are shown in Figure 7.

**Figure 8:**
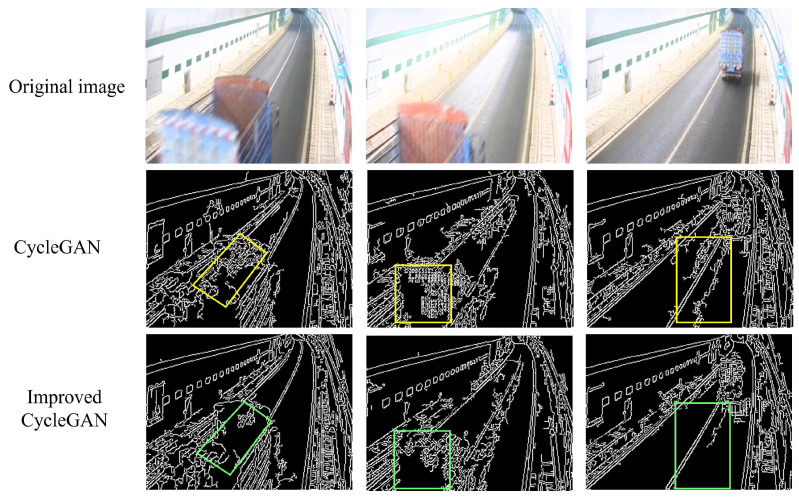
These are the edge detection results of the generated images in the experiment.

The edge detection results of the generated images in our experiment are presented in Figure 8. Comparing the edge detection images of the original CycleGAN-generated images (highlighted in yellow boxes) with those generated by our model (highlighted in red boxes), it is evident that integrating edge loss enables the model to accurately capture vehicle edge information from the original images while reducing environmental noise artifacts along edges. For example, in the second case shown in Figure 8, CycleGAN introduces excessive edge noise around vehicles, whereas the improved CycleGAN incorporating edge loss produces vehicle edges that closely align with those in the original image.

#### 4.4.2. Validation of the Adaptive Vehicle Detection Model

To validate the effectiveness of the framework of our model, we conducted two experimental setups. Initially, we trained the YOLOv7 model using the dataset containing different luminance scenarios. Subsequently, we applied the adaptive luminance adjustment module to the dataset and retrained the YOLOv7 model with the adjusted data.

**Figure 9:**
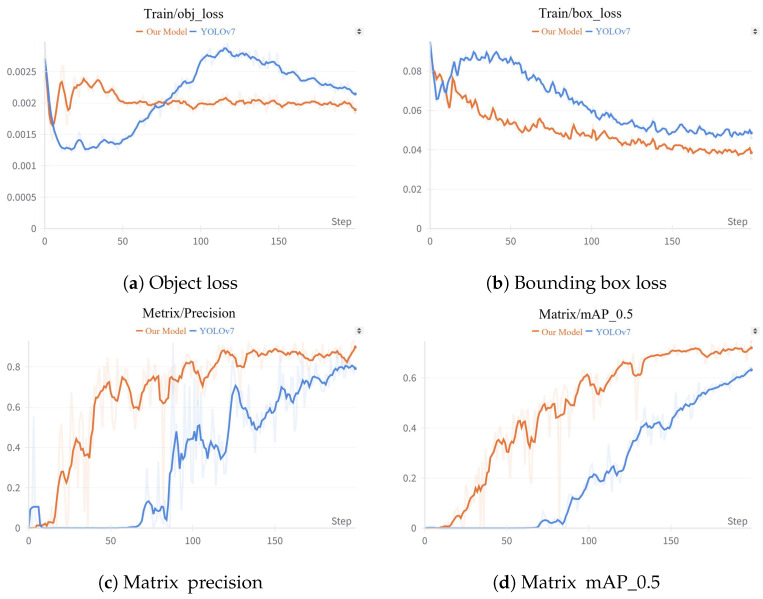
The loss during the model training. (**a**) Object loss during the model training. (**b**) Bounding box loss during the model training. (**c**) The precision during the model training. (**d**) The mAP_0.5 during the model training.

The training processes for both experimental setups are shown in Figure 9. The object loss and bounding box loss gradually decreased, converging as the number of iterations approached 200. The results shown in Figure 9 indicate that the vehicle detection model incorporating the adaptive luminance adjustment module achieved faster training convergence and lower loss function values, thereby demonstrating the effectiveness of our approach.

The vehicle detection metrics of our model compared to YOLOv7 on the test dataset are shown in Table 2. The results indicate that, after incorporating the adaptive luminance adjustment module, our model achieved a 16.3% improvement in Precision, a 1.7% improvement in Recall, and a 9.8% improvement in mAP_0.5 compared to the original YOLOv7. These findings demonstrate that our model effectively enhances the accuracy of visual vehicle detection algorithms under abnormal luminance scenarios.

**Figure 10:**
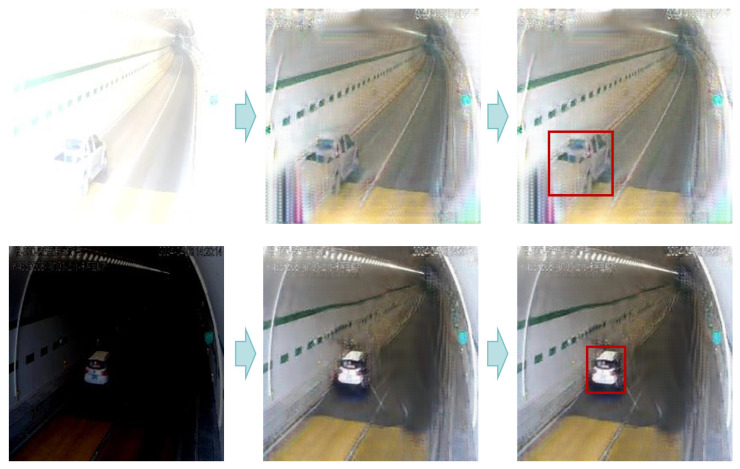
The visualization results of vehicle detection. The red box in the image represents the vehicle detection result.

Figure 10 illustrates the visualization results of vehicle detection using our model. Initially, our model adaptively adjusts the luminance intensity of abnormal images, thereby enhancing their information quality. Subsequently, the vehicle detection module accurately detects vehicle positions.

#### 4.4.3. Validation of Model Scalability

To validate the scalability of our model, experiments were conducted with Fast R-CNN and SSD. These experiments trained models with and without an adaptive luminance adjustment module on a dataset specifically designed for vehicle detection under abnormal luminance scenarios. Table 3 displays the vehicle detection metrics on the test dataset. Models using the adaptive luminance adjustment module are labeled as Fast R-CNN+ and SSD+. The results show improvements across all vehicle detection metrics when the adaptive luminance adjustment module was used during training, indicating enhanced capability in capturing vehicle information under abnormal luminance scenarios.

## 5. Conclusions

Mitigating the impact of abnormal luminance scenarios on vehicle detection model performance is crucial for achieving accurate results. Introducing an adaptive luminance adjustment module to adjust image lighting enhances the accuracy of vehicle detection models. This approach aligns with current trends in object detection under challenging luminance conditions.

While previous studies using Fast-RCNN and YOLO have demonstrated the powerful feature extraction capabilities of deep learning methods, there remain challenges regarding low detection accuracy in specialized environments. This issue primarily arises from the direct impact of such environments (e.g., abnormal luminance scenarios) on image pixels, altering the feature distribution captured by vehicle detection models and thereby hindering their performance.

This paper proposes an adaptive vehicle detection model to address the challenges posed by abnormal luminance in real tunnel scenarios from a novel perspective. Our model utilizes an enhanced CycleGAN to learn the distinctive features between abnormal and normal luminance scenarios, enabling effective luminance adjustment in images affected by irregular lighting conditions. In experiments conducted on our collected abnormal luminance dataset, the proposed vehicle detection model achieved a 16.3% improvement in Precision, a 1.7% improvement in Recall, and a 9.8% improvement in mAP_0.5 compared to the original YOLOv7. This indicates that the proposed method significantly enhances the accuracy of vehicle detection models under these challenging conditions.

Additionally, by integrating CycleGAN with an edge loss function, our improved CycleGAN achieved a 7.268% decrease in FID score for adjusting luminance from high to normal intensity and a 3.540% decrease for adjusting from low to normal intensity. As observed in the edge detection images of the generated pictures, the model effectively preserves the original edge information of images during generation, preventing the introduction of redundant edge details and thereby enhancing the quality of the generated images.

Moreover, we replaced YOLOv7 in the framework with other vehicle detection models (Fast-RCNN and SSD). Experiments show that the proposed vehicle detection framework effectively enhances the performance of vehicle detection models. This indicates that the adaptive luminance adjustment module is versatile and can be applied to other vehicle detection frameworks.

Despite the advantages of our method in vehicle detection tasks under abnormal luminance, there is still room for further improvement. Future research can focus on the following aspects: Firstly, exploring additional scenarios for applying generative models combined with object detection models to verify the method’s generality and adaptability. Secondly, while adding edge loss can effectively preserve edge information in original images, some edges completely obscured by abnormal luminance, such as vehicles hidden by strong light, are challenging to regenerate. Future research could explore integrating contextual information from continuous image sequences to enable the model to capture edge details lost under abnormal luminance scenarios, thereby enhancing the practicality and efficiency of current methods.

## Figures and Tables

**Table 1:** These are the evaluation results of the FID metric for the generative models.

Model	High to Normal (FID)	Low to Normal (FID)
CycleGAN	30.242	199.342
Improved CycleGAN	28.044 ↓	192.285 ↓

The downward arrow ↓ indicates an improvement in the FID score.

**Table 2:** The vehicle detection metrics of YOLOv7 and our model (YOLOv7+).

Model	Precision	Recall	mAP_0.5
YOLOv7	0.790	0.611	0.640
YOLOv7+	0.953 ↑	0.628 ↑	0.738 ↑

The up arrow ↑ indicates an improvement in the detection metrics.

**Table 3:** The vehicle detection metrics of models on the test dataset.

Model	Precision	Recall	mAP_0.5
Fast-RCNN	0.632	0.602	0.625
Fast-RCNN+	0.676 ↑	0.628 ↑	0.653 ↑
SSD	0.618	0.582	0.593
SSD+	0.629 ↑	0.594 ↑	0.609 ↑

The up arrow ↑ indicates an improvement in the detection metrics.

## Data Availability

The data presented in this study are available on request from the corresponding author. The data are not publicly available due to confidentiality issues.
